# Progressive Training in Laparoscopic Suturing: From Motor Coordination to Intracorporeal Suturing Within Four Hours

**DOI:** 10.7759/cureus.100126

**Published:** 2025-12-26

**Authors:** Rodrigo Sanderson, Ricardo Ribeiro Correa Filho, Lucas Godoy Dias Sanderson, José Sebastião Dos Santos

**Affiliations:** 1 Surgery and Anatomy, Hospital das Clínicas da Faculdade de Medicina de Ribeirão Preto, Universidade de São Paulo, Ribeirão Preto, BRA; 2 Surgery, Faculdade de Ciências Médicas da Santa Casa de São Paulo, São Paulo, BRA; 3 Surgery and Anatomy, Hospital das Clinicas da Faculdade de Medicina de Ribeirão Preto, Universidade de São Paulo, Ribeirão Preto, BRA

**Keywords:** artificial intelligence, medical education, motor coordination, square knot, suture teaching, virtual simulation

## Abstract

The teaching of basic suturing and knot-tying techniques requires a structured method for the development of these skills, and acquiring such competence is essential in surgical training. The objective of this article is to present a structured four-hour program of progressive training based on principles of motor coordination, spatial cognition, and mastery of the non-dominant hand, focusing on gradual technical construction. The method includes specific exercises, such as the dancing needle, C-loop and D-loop formation, needle-passing practice, and the performance of intracorporeal sutures using physical simulators. The process preserves traditional pedagogy centered on manual skills, but it is designed in a modular format for future integration with virtual simulation platforms and artificial intelligence. This model provides an efficient approach to accelerate the initial technical development in laparoscopy and has the potential to be incorporated into contemporary surgical education programs that employ immersive learning methods.

## Introduction

The teaching of basic knot-tying and suturing techniques is fundamental in surgical training, as it is crucial for technical success and patient safety [1,2]. Traditionally, manual skills are taught in simulated environments, emphasizing repetition of movements and systematic correction of errors [3]. However, the pedagogical approach adopted during initial training can significantly influence the quality of complex motor skill acquisition.

Inspired by the method used in tennis training -- where movements are progressively developed until the complete execution -- the present study proposes the application of analogous principles to surgical education. Surgical training, much like sports training, benefits from the gradual development of skills, with an emphasis on fine motor coordination, spatial cognition, control of the non-dominant hand, and deliberate repetition [4,5].

Recently, the relevance of simulation-based practices and enhanced feedback for improving technical training has been highlighted [6,7]. Virtual simulation models and the use of artificial intelligence (AI) have advanced rapidly, enabling objective performance assessment and personalized training [8-10]. Despite the growth of these technologies, it remains essential that early training continues to be grounded in solid principles of motor learning, which are crucial for a safe transition between analog and digital training [11].

The incorporation of structured simulation stations into the surgical education curriculum facilitates the acquisition of psychomotor skills and promotes the transfer of skills to the clinical environment, as evidenced by both high-fidelity simulators and accessible, low-cost modules. These findings corroborate the growing need to integrate advanced simulation models and assessments into surgical training programs to improve the teaching of laparoscopic suturing [12].

The purpose of this article is to present a systematic framework for teaching surgical sutures and knots, based on gradual motor progression, through structured practical exercises, specific needle-handling techniques, and the conscious construction of the square knot in order to align the foundational principles of manual skill development with those applied in contemporary virtual simulation platforms.

## Technical report

The training is conducted weekly at the Experimental Surgery Laboratory of the Ribeirão Preto Medical School, University of São Paulo-FMRP-USP and involves first-, second-, and third-year residents (R1, R2, and R3) from the General Surgery program. The facility is equipped with three multi-perforated black boxes, each containing a 0° full HD optical system with manual focus and 180-degree movement capability. The instruments employed include Maryland forceps, a grasper, scissors, and a needle holder.

The laboratory setup comprises a multi-perforated training platform featuring pins and plastic rings of different colors, rubber glove cuffs for strength training, a 30 cm cord for coordination exercises, and a silicone pad that simulates real tissue conditions during laparoscopic procedures.

The training adopts a progressive approach, beginning with basic motor coordination exercises and advancing toward more complex suturing and knot-tying techniques.

Pedagogically, the program is structured in the following stages: (1) Motor coordination and instrument familiarization: Residents begin with exercises designed to develop motor coordination and familiarity with laparoscopic instruments. Using the multi-perforated training platform, they perform tasks that involve moving forceps through pins and colored rings, thereby promoting manual dexterity and movement precision; (2) Force control and precision: Subsequently, residents practice force control using rubber glove cuffs, which provide variable resistance and simulate tissue tension encountered in real procedures. This stage is essential for preventing tissue damage during surgery; (3) Coordination exercises with cord: A 30 cm cord is used to perform coordination exercises, enabling residents to develop manipulation and control skills by operating instruments in different directions and depths; (4) Training with silicone pad: The silicone pad is introduced as an advanced training module, providing a more realistic experience by replicating conditions found in laparoscopic procedures. Its surface contains continuous incisions at various angles, allowing for the practice of knot-tying and precise needle-holder manipulation; (5) Needle handling and positioning: Residents learn to properly position and handle the needle to facilitate its passage through simulated tissues. This stage focuses on developing the ability to position the needle within the needle holder at different angles, a skill essential for effective suturing; (6) Depth and penetration: Practicing needle insertion at varying depths is fundamental to ensuring suture efficacy. These exercises are conducted on simulated tissues, allowing residents to adjust both the force and the angle of needle penetration; (7) Suturing at different angles and positions: Residents are trained to adapt suturing techniques to various anatomical orientations and locations. This includes practicing sutures at different angles and positions, using models that simulate real conditions encountered during laparoscopic procedures; and (8) Surgical knot formation and suturing: The final stage of the training involves the formation of surgical knots, during which the medical residents learn to apply the appropriate amount of force during suturing to avoid tissue damage. This practice is carried out on simulated models, allowing for repetition and refinement of the technique. After the introduction of the general principles of the progressive method, the training is structured in sequential stages, each aimed at developing specific skills necessary for the technical performance of laparoscopic sutures. Below, each phase of the training process is described in detail, along with the suggested duration for each stage of the exercise.

Initial training in motor coordination, depth cognition, and non-dominant hand (15 minutes)

The training begins with the development of motor coordination and familiarization with laparoscopic instruments, using a multi-perforated training platform equipped with colored pins and rings. The medical residents perform basic movements, including levering, translation (back and forth), rotation, and opening and closing of forceps, with the goal of promoting manual dexterity and motor precision. The activity requires the use of the Maryland dissector and the grasper for positioning the rings, exploring different entry angles through the controlled rotation of the Maryland. Throughout the exercise, residents must keep both hands within the monitor’s visual field, continuously synchronizing their movements. Subsequently, the focus shifts to training depth cognition, using spatial perception to place the rings at different heights and depths, simulating the manipulation of internal structures during laparoscopic surgery (Video 1). Practicing with the non-dominant hand is integrated into the protocol, challenging residents to perform precision and positioning tasks with this hand, thereby promoting the development of ambidexterity -- a critical skill for the efficient execution of minimally invasive procedures.

**Video 1:** Motor coordination, depth cognition, and non-dominant hand exercise. Exercise demonstrating non-dominant hand technique training while passing the ring back and forth across the field, always keeping both forceps in the center of the screen.

Coordination and traction training with marked cord (20 minutes)

The second exercise consists of training coordination and traction force using a cord approximately 30 cm long, marked at regular 2 cm intervals. In the first stage (10 minutes), the medical residents use the Maryland dissector and the grasper to pull the cord in alternating movements with the right and left hands, following the markings (Video 2). This exercise aims to develop bimanual coordination and fine force control with laparoscopic instruments. In the second stage (10 minutes), the cord is positioned close to the laparoscopic camera and is tensioned by the forceps holding alternating markings. A continuous traction movement is then performed, simulating the “accordion effect” of the thread, with the goal of training the removal of the surgical thread memory and optimizing the sliding during intracorporeal suturing (Video 3).

**Video 2:** Pull the cord in alternating movements with the right and left hands. Demonstration of coordinated traction between the two forceps on the marked cord, highlighting fine movements and the maintenance of both forceps near the center of the image.

**Video 3:** Exercise to removal of the surgical thread memory. Demonstration of alternating thread pulling movements with both forceps.

Force-control and coordination exercises with elastic bands in pins (15 minutes)

In this exercise, medical residents use rubber bands made from the cuffs of surgical gloves, attached to pins on a multi-perforated training platform, to practice force control and fine motor coordination. One end of the elastic band is fixed to a stable point at the top of the platform, while the other end is attached to one of the pins (Video 4). The goal is to perform traction and counter-traction movements without displacing the base of the platform, simulating the natural tissue resistance encountered during laparoscopic surgery. This exercise develops tactile sensitivity and control of applied force, critical skills for safely performing intracorporeal sutures and dissection maneuvers.

**Video 4:** Force-control and coordination exercises with elastic bands in pins. Demonstration of the rubber band traction by one of the graspers during counter-traction applied by the contralateral hand to perform the proposed movement.

Needle positioning training and dancing needle control (augmented feedback-20 minutes)

At this stage, medical residents develop precise needle positioning and manipulation skills using the dancing needle concept. Initially, training is performed outside the simulator (augmented feedback), focusing on motor memory development before practical application in the simulator (normal feedback).

The dancing needle consists of using the suture thread to create a controlled glide of the needle over the Maryland grasper, with its concavity facing upward. The resident must learn to control this gliding movement, keeping the needle axis aligned and avoiding falls or deviation, which is essential to correctly mount the needle on the needle holder (Video 5). The manipulation requires subtlety and fine bimanual coordination.

**Video 5:** Dancing needle control. On the left, the needle holder maintains traction on the thread, assisting proper positioning for needle grasping; on the right, note the assembly of the needle grip at the desired angle.

For proper backhand positioning, the thread is pulled backward and downward relative to Maryland, favoring the correct orientation of the needle for reverse grip. It is emphasized that the needle should be visually divided into three parts: distal two-thirds and one proximal third. The ideal grasp is achieved when the needle holder is positioned precisely at the transition between the distal two-thirds and the proximal third. Inserting the needle too deeply into the holder impairs dexterity and limits maneuverability during suturing. The grip must be firm yet gentle, ensuring control without compromising the freedom of movement necessary for the rotation of the needle in the different tissue planes.

Practical mounting of forehand and backhand needles in the simulator (30 minutes)

After developing initial motor memory, residents progress to practice mounting the needle on the laparoscopic simulator, applying forehand and backhand techniques.

In the forehand position, the needle is mounted with its tip facing the surgeon, facilitating direct passage through the target tissue. The Maryland grasper and needle holder are used in synchrony: the needle holder grips the thread near the needle, while the Maryland grasper, with its concavity facing upward, “fishes” the needle. The grasp should occur between the distal two-thirds and proximal third of the needle, avoiding excessive insertion into the holder to maintain dexterity and fine control (Video 6).

**Video 6:** Forehand positioning. Exercise for the positioning of the needle grip in the forehand configuration.

The dancing needle technique is incorporated at this stage, where the needle is manipulated using the thread, allowing adjustment of its orientation before final grasping. This exercise develops control of gliding movement and spatial perception of the suture trajectory.

In the backhand position, the needle is mounted with its tip facing away from the surgeon. To achieve this orientation, the resident must pull the thread backward and downward relative to the Maryland, promoting appropriate needle rotation and ensuring correct loop formation (Video 7).

**Video 7:** Backhand positioning. Exercise for the positioning of the needle grip in the backhand configuration.

These exercises are practiced repeatedly, alternating forehand and backhand configurations to reinforce mastery of instrument handling, bimanual coordination, and precision in entry angles.

Training with the silicone pad (30 minutes)

The silicone pad is used to simulate realistic tissue conditions during laparoscopic procedures. Activities are organized as follows: Symmetrical needle passage at entry and exit at the base position: Residents practice accurate, symmetrical needle insertion and exit to ensure correct stitch placement. The sequence is divided into four movements: (1) symmetrical needle passage, elevating the tip upon exit and penetrating tissue at a 90° angle; (2) grasping the needle tip with the Maryland grasper; (3) releasing the needle holder, which is now closed and should be positioned under the needle base to provide proper tissue support; (4) controlled traction of the needle toward the portal, allowing a new grasp by the needle holder in the correct position. Important: needle traction must always remain within the monitor’s visual field (Video 8).

**Video 8:** Training with the silicone pad. Exercise of the four-step needle passage sequence.

In optimization of needle withdrawal movements, residents develop specific maneuvers for efficient needle removal from tissue, focusing on maintaining field stability and preparing for the next needle setup to optimize procedural flow.

Incision angle training (15 minutes)

After achieving initial motor fluency, residents practice needle passage using forehand and backhand techniques to refine incision angle precision.

During backhand passage, the thread is pulled backward and downward, aligning the needle parallel to the Maryland grasper, improving stability and readiness for the next movement.

In the backhand technique, counter-traction is applied with the needle holder at the needle base, near the tissue exit point. The Maryland grasper moves toward the opposite side of the trocar, enabling control of the force vector applied to the tissue (Video 9).

**Video 9:** Incision angle training. Suturing exercise in forehand and backhand formatting.

This training aims to consolidate precision in the needle’s entry and exit vectors, key factors for safe and efficient laparoscopic suturing.

Knot construction-“C-loop” and “D-loop” techniques-outside simulator (augmented feedback)-15 minutes

**Figure 1:**
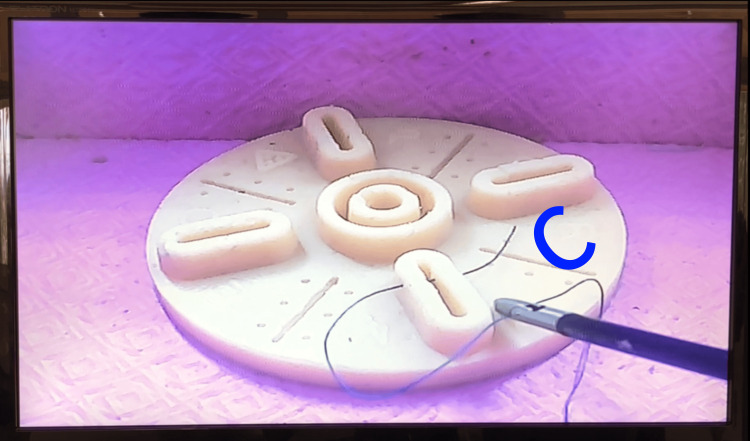
C loop. Schematic representation of C-loop formation.

Surgical knot formation is taught using the C-loop and D-loop techniques. The steps include: Forming the “C”: Residents form the letter “C” in front of the base position, with both graspers (Maryland and needle holder) in a cupped position, that is, concavity facing downward (Figure 1). The right-hand grasper captures the suture thread, while the left-hand grasper passes through the “C.” Next, the right-hand grasper rotates over the left, forming the first loop of the knot. Both hands follow the path of the free suture end (the short segment after needle passage), and the left-hand grasper captures this segment. The knot is then adjusted according to the original needle entry direction.

**Figure 2:**
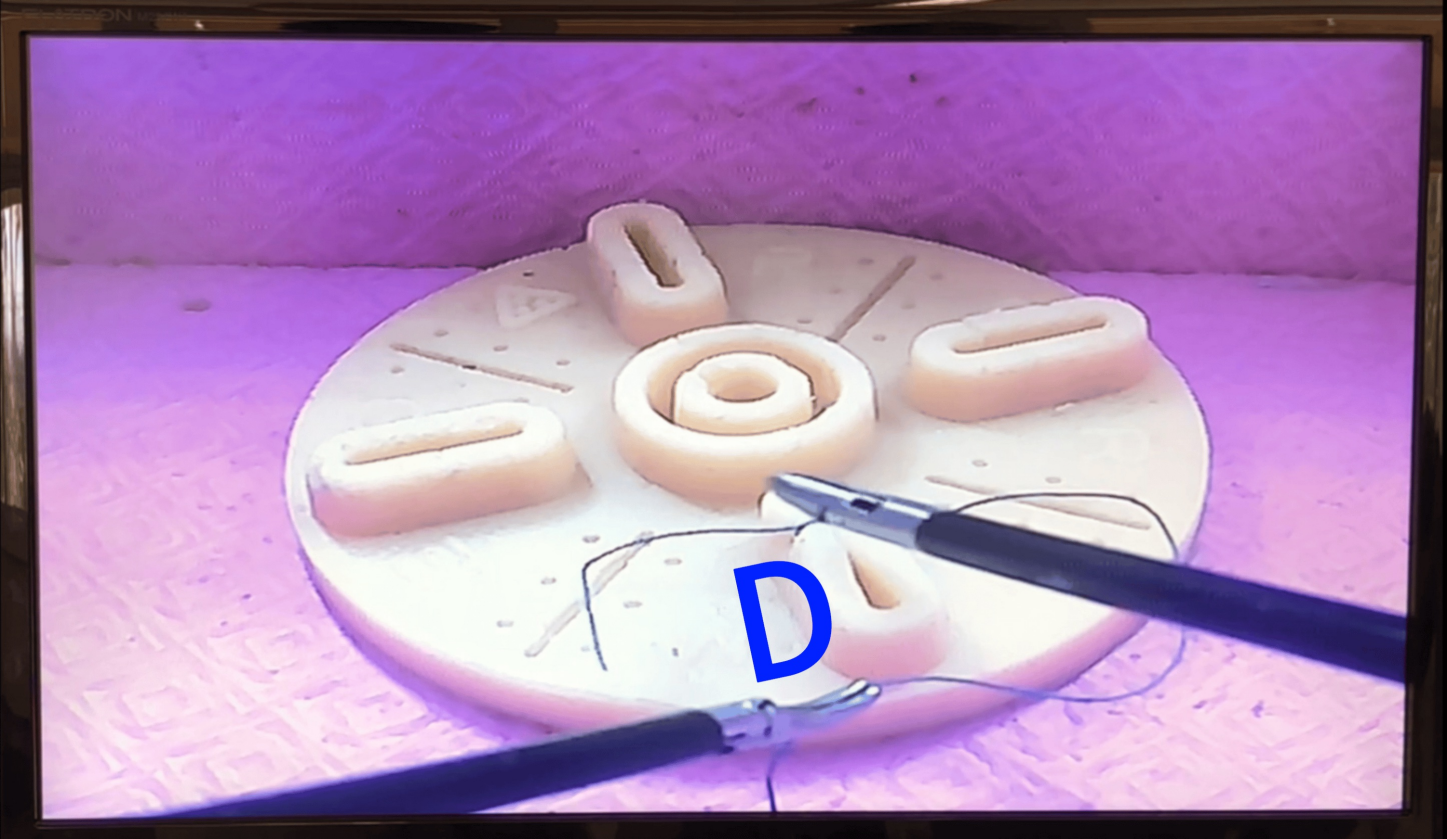
D loop. Schematic representation of D-loop formation.

Forming the “D”: After tightening the first half-knot, the right-hand grasper remains fixed on the suture thread. The left-hand grasper captures the thread beneath the right-hand one, forming the D loop (Figure 2). Then, the right-hand grasper advances inside the “D” and remains static, while the left-hand grasper, holding the thread, rotates over the dominant hand to adjust the next half-knot. Again, both hands follow the path of the free suture end (the short segment after needle passage), and the right-hand grasper captures this segment.

Suture application in the simulator (40 minutes)

Residents apply the techniques learned during augmented feedback and now practice under normal feedback conditions, performing exercises that include: ten stitches per side using “C” and “D” techniques. Performing sutures with ten stitches on each side to practice consistency and precision (Video 10).

**Video 10:** Suture application in the simulator This exercise demonstrates the practice of all previously trained techniques, aiming at performing laparoscopic suturing in all its steps.

Suturing with two glove fingers in inclined position (40 minutes)

Using two glove fingers fixed parallel to each other in an oblique position, secured with pins to the base and bottom of the simulator, residents perform a suture of approximately three to five stitches, simulating tissue approximation (Video 11). At the end, a short suture loop is created, serving as the base for the final knot closure.

**Video 11:** Suturing with two glove fingers in an inclined position. This exercise presents greater technical difficulty due to the inclination and sensitivity of the tissue, representing anatomical structures for laparoscopic suturing training.

## Discussion

The development of fundamental laparoscopic skills is recognized as an essential component of contemporary surgical training. The training protocol was structured progressively and based on validated surgical education practices, adhering to international guidelines and the recommendations of the Society of American Gastrointestinal and Endoscopic Surgeons (SAGES) [13,14].

Initial training focused on motor coordination, depth perception, and controlled instrument movement, which is critical to overcoming the limitations of two-dimensional vision and the hand-eye dissociation characteristic of laparoscopic surgery. Exercises using multi-perforated platforms and rings reinforce active bimanual use and movement precision, establishing the foundation for more complex manipulations [15].

The introduction of exercises involving marked cords and elastic bands broadens the range of trained skills, simulating the variable resistance of living tissues, and promoting the development of tactile sensitivity. According to SAGES guidelines, practices that incorporate force control and sensory feedback are essential to minimize complications such as inadvertent lacerations or perforations [16].

Needle manipulation techniques -- including dancing needle training and forehand and backhand mounting -- emphasize the importance of precision in instrument handling. Controlling the needle with the needle holder positioned between the distal two-thirds and the proximal third prevents the loss of manual dexterity. Practicing proper needle mounting, maintaining visual control during thread traction, and coordinating Maryland grasper positioning for subsequent passages are key steps toward motor fluency, a prerequisite highlighted by SAGES before progressing to more advanced simulations [13].

Training on the silicone pad provides a simulated environment with elasticity and resistance similar to human tissue [17]. The detailed sequence of movements -- needle entry at a 90-degree angle, controlled tip grasping, repositioning of the needle holder, and progressive traction -- reinforces precision and maintenance of the visual field, optimizing both needle passage and subsequent suture execution [18].

The construction of intracorporeal knots using the C-loop and D-loop techniques was carefully integrated into the curriculum. In forming the C, the resident works the loop in front of the fixed end of the thread, with the dominant grasper rotating over the non-dominant one. For the D, after the initial adjustment, the thread end is rotated in a controlled manner over the dominant grasper, consolidating the knot.

Finally, the suturing exercise using parallel glove fingers fixed at an inclined angle simulates the real need to approximate tissues under controlled tension. The creation of a short loop at the end of the stitch sequence facilitates the final knot, a widely recommended practice to ensure the safety of laparoscopic anastomoses and closures.

The training protocol described here follows the philosophy of deliberate repetition and progressive complexity, factors proven effective for the acquisition of long-lasting psychomotor skills [4]. Furthermore, it aligns with SAGES principles for simulator validation, emphasizing that basic motor fluency is indispensable before advancing to complex simulation scenarios or live models [13].

The implementation of this program contributes not only to the acquisition of technical competence among surgical residents but also to the development of situational awareness, ergonomic handling, and safe surgical decision-making, cornerstones in the education of surgeons who perform laparoscopic procedures with excellence.

## Conclusions

The laparoscopic training protocol described in this study establishes a structured, competency-based progression aligned with international guidelines for the development of psychomotor skills in minimally invasive surgery. The proposed exercises integrate bimanual coordination practice, precise needle manipulation, intracorporeal knot construction, and suturing under simulated realistic tissue resistance.

Given the importance of scientific validation for educational methods, a future study is planned to formally evaluate this protocol. Validation will be conducted through objective metrics of technical performance (execution time, knot accuracy, and quality) and subjective analysis of participants' experience, using standardized questionnaires, in accordance with the recommendations of the Society of American Gastrointestinal and Endoscopic Surgeons (SAGES).
